# Inter-task transfer of force gains is facilitated by motor imagery

**DOI:** 10.3389/fnins.2023.1228062

**Published:** 2023-08-14

**Authors:** Eric Piveteau, Franck Di Rienzo, Olivier Bolliet, Aymeric Guillot

**Affiliations:** Inter-University Laboratory of Human Movement Biology-EA 7424, University of Lyon, University Claude Bernard Lyon 1, Villeurbanne, France

**Keywords:** movement imagery, inter-task transfer, motor performance, force, mental practice

## Abstract

**Introduction:**

There is compelling evidence that motor imagery (MI) contributes to improve muscle strength. While strong effects have been observed for finger muscles, only few experiments with moderate benefits were conducted within applied settings targeting large upper or lower limb muscles. The aim of the present study was therefore to extend the investigation of embedded MI practice designed to improve maximal voluntary strength on a multi-joint dynamic exercise involving the lower limbs. Additionally, we tested whether targeting the content of MI on another movement than that physically performed and involving the same body parts might promote inter-task transfer of strength gains.

**Methods:**

A total of 75 participants were randomly assigned into three groups who underwent a physical training on back squat. During inter-trial recovery periods, a first MI group (*n* = 25) mentally rehearsed the back squat, while a second MI group (*n* = 25) performed MI of a different movement involving the lower limbs (deadlift). Participants from the control group (*n* = 25) completed a neutral cognitive task during equivalent time. Strength and power gains were assessed ecologically using a velocity transducer device at 4 different time periods.

**Results:**

Data first revealed that participants who engaged in MI of the back squat improved their back squat performance (*p* < 0.03 and *p* < 0.01, respectively), more than the control group (*p* < 0.05), hence supporting the positive effects of MI on strength. Data further supported the inter-task transfer of strength gains when MI targeted a movement that was not physically trained (*p* = 0.05).

**Discussion:**

These findings provide experimental support for the use of MI during physical training sessions to improve and transfer force development.

## 1. Introduction

Motor imagery (MI) refers to the brain capacity to covertly simulate actions without engaging in their overt execution. It is now well-established that MI contributes to enhance motor learning and performance (for an overview, see Guillot and Collet, 2008; Schuster et al., 2011), and promote motor recovery in populations of patients or injured athletes (Di Rienzo et al., 2014; Zhao et al., 2022). Spurred by its effects on movement accuracy, speed and efficacy, MI has gained popularity among athletes and coaches and is currently considered a “*Centre pillar of applied sport psychology*” (Morris et al., 2004, p. 344; Cumming and Williams, 2012). A handful of experimental studies provided evidence that MI can improve force elicited by voluntary contractions (for reviews, see Slimani et al., 2016; Paravlic et al., 2018; Liu et al., 2023). In a pioneering study, Cornwall et al. (1991) provided evidence that 30 min of MI practice increased isometric strength of the quadriceps by 16%. This pattern of positive effects was later replicated in a series of MI interventions targeting upper and lower limb muscles, with force gains ranging from 5 to 31% (e.g., Yue and Cole, 1992; Smith et al., 2003; Zijdewind et al., 2003; Ranganathan et al., 2002, 2004; Sidaway and Trzaska, 2005; Bahari et al., 2011; Darvishi et al., 2013; Jiang et al., 2017; Grosprêtre et al., 2018; Scott et al., 2018). While MI can contain elements referring to all sensory modalities, data revealed that higher force gains were elicited after kinesthetic and first-person visual imagery compared to third-person visual imagery (Yao et al., 2013). Functional magnetic resonance imaging studies further provided evidence that kinesthetic MI yields a stronger activation of brain motor networks including primary motor and premotor cortices, supplementary motor area as well as basal ganglia and cerebellar regions, compared to visual modalities (Solodkin et al., 2004; Guillot et al., 2009), hence having greater potential to leverage experience-based neural plasticity promoting performance enhancement. Interestingly, acute force gains were also observed after a single session of embedded MI training, suggesting short-term changes in the cortical gain over motor units (Di Rienzo et al., 2015; Dos Anjos et al., 2022).

Force improvements induced by MI training stem from neural adaptations. First, there is recent evidence for a downregulation in central inhibition improving motor unit recruitment and synchronization (Grosprêtre et al., 2019). MI training also allows the brain to generate stronger motor command signals to the muscle and recruit motor units that are inactive in an untrained state, and/or drive the active motor units to higher intensity (Ranganathan et al., 2004). Although this remains a working hypothesis, MI is thus expected to either facilitate motor unit recruitment, promote synchronization, or increase firing rate. This postulate is consistent with the fact that the effects of MI on force were primarily observed for muscles that exhibit large cortical representations in the sensorimotor homunculus (Liu et al., 2023). Di Rienzo et al. (2015) provided behavioral evidence that implementing MI (either activating or relaxing MI) during the recovery period of physical training primed neural excitability within task-specific somatic pathways, yielding improved maximal isometric force performance through enhanced muscle activation and intermuscular coordination. By examining the corticomotor plasticity elicited by MI of maximal isometric contractions, a recent electroencephalographic study by Dos Anjos et al. (2022) suggested that the priming effects of MI on force were underpinned by short-term modulations in agonist/antagonist co-activation. These mirror short-term neural adaptations elicited during the early stages of resistance training. Overall, there is a consensus that force gains induced by MI practice result from cortical remapping. These occur in the absence of muscle hypertrophy or any other adaptation of muscle structure and morphology such as myofibrillar growth or proliferation (Yue and Cole, 1992).

Most research on the effects of MI on force performance focused on maximal isometric contractions (Liu et al., 2023). Few authors questioned the effects of MI on complex polyarticular movements involving the whole body or large muscle groups against movable resistance. To support the value of using MI in an integrated form, studies have shown that using MI during the break from physical training is not harmful and does not add physical or mental fatigue (Rozand et al., 2014). In a pioneer experiment, Lebon et al. (2010) questioned the benefits of embedded MI during inter-set periods of weightlifting training. Over a 6 weeks training period including 12 MI sessions, they reported low effect size force improvements for the leg press, but not the bench press. Reiser et al. (2011) later tested the magnitude of strength gains after a high-intensity resistance MI training on four classical force exercises (bench pressing, leg extension, triceps extension, and calf raising). Interestingly, they looked for the selective effects of different combinations of physical and MI training. While strength gains observed in the MI groups were lower than those achieved by the physical training group, regardless of the MI to physical practice ratio, the authors concluded that MI might be a promising supplementary method for improving muscle strength. This would particularly be true for finger muscles (Liu et al., 2023). Although there are only limited results available on the effects of MI on force performance in polyarticular movements, preliminary data supported that MI could be considered an adjunct to resistance training programs. Further experimental investigation is however required before drawing firm conclusions and practice guidelines. Also, why MI yields stronger effects for some movements/muscles than others remains uncertain. Admittedly, this could be due to confounding factors such as motivation to achieve success, and individual commitment and accomplishment to certain exercises. Also, there is no proof of long-lasting effects of MI on strength for polyarticular and dynamic movements, which is a limiting factor to the allocation of practitioners’ time and resources in prescribing MI of muscle contraction during inter-trial resting periods of their training sessions. At this stage, a working hypothesis is that MI may be primarily useful as an acute application for force performance such as a 1-repetition maximum (1RM) performed by a confirmed athlete.

An exciting issue, however, is whether MI practice of a secondary polyarticular movement distinct from that trained physically, but involving the same agonist muscles, could prime the transfer of force gains. Surprisingly, the scientific literature paid little attention to transfer effects resulting from MI practice (Guillot et al., 2022). The most studied form of transfer is contralateral force gains after unilateral training, which reflects a cross-education or sparing effect (Andrushko et al., 2018; Green and Gabriel, 2018a,b; Manca et al., 2021). Accordingly, a motor skill trained on one body side may also lead to improvement in the untrained side (Carroll et al., 2006; Lee and Carroll, 2007). This represents a first class of transfer effects, which we will qualify here as *intra-task* transfer. The putative role of MI training on intra-task transfer has already been observed (e.g., Yue and Cole, 1992; Bouguetoch et al., 2021). However, whether MI training could facilitate *inter-task* transfer, i.e., facilitate generalization of force gains to distinct polyarticular movements than those trained physically, remains unknown. Such transfer of strength and power training has been extensively demonstrated during physical practice training programmes, as a result of intermuscular coordination (Young, 2006). Although not systematic (Roure et al., 1998), there is few evidence for similar inter-skill transfer effects as a result of MI interventions in the motor learning literature (Lejeune et al., 1994; Nicholson et al., 2018; Guillot et al., 2022). In these studies, which did not investigate force performance, benefits provided by a MI intervention were contributed to improve performance of another motor skill. This was particularly true when MI was performed on a complex motor task and when specifically focusing attention and emphasizing the corresponding intention to the transfer of a skill during the MI exercises (Guillot et al., 2022).

The aim of the present study was to address two gaps in the scientific literature. A first objective was to provide further scientific investigation of the effects of MI training on force performance from dynamic polyarticular movements. Particularly, we sought to investigate whether implementing MI within applied setting improved force performance on the most common powerlifting exercises. We hypothesized that engaging in a MI force training programme might contribute to improve force of the corresponding targeted movement. A secondary aim was to test whether implementing MI training during the inter-trial periods of another dynamic polyarticular exercise to that physically trained, which shared common agonists, might elicit inter-task force transfer effects. We postulated that MI might promote such inter-task force transfer by further increasing the performance of the secondary movement that was trained only through MI.

## 2. Materials and methods

### 2.1. Participants

A total of 75 participants (36 women and 39 men, mean age = 28.43 ± 6.33 years) volunteered to participate in the present experiment. All were CrossFit^®^ athletes engaged in competitive activities at the national level. Participants had no former experience of MI training to improve force performance, and were subjected to two MI familiarization sessions before the experiment. Participants were also screened based on their ability to engage appropriately in MI practice using the movement imagery questionnaire-3f (MIQ-3f; Robin et al., 2020). There was no other exclusion criteria, and no information concerning the purpose of the study was provided until completion of the design. The local ethics committee approved the experiment, and participants’ written consent was obtained according to the statements of the Declaration of Helsinki (1982).

### 2.2. Experimental design

**Figure 1:**
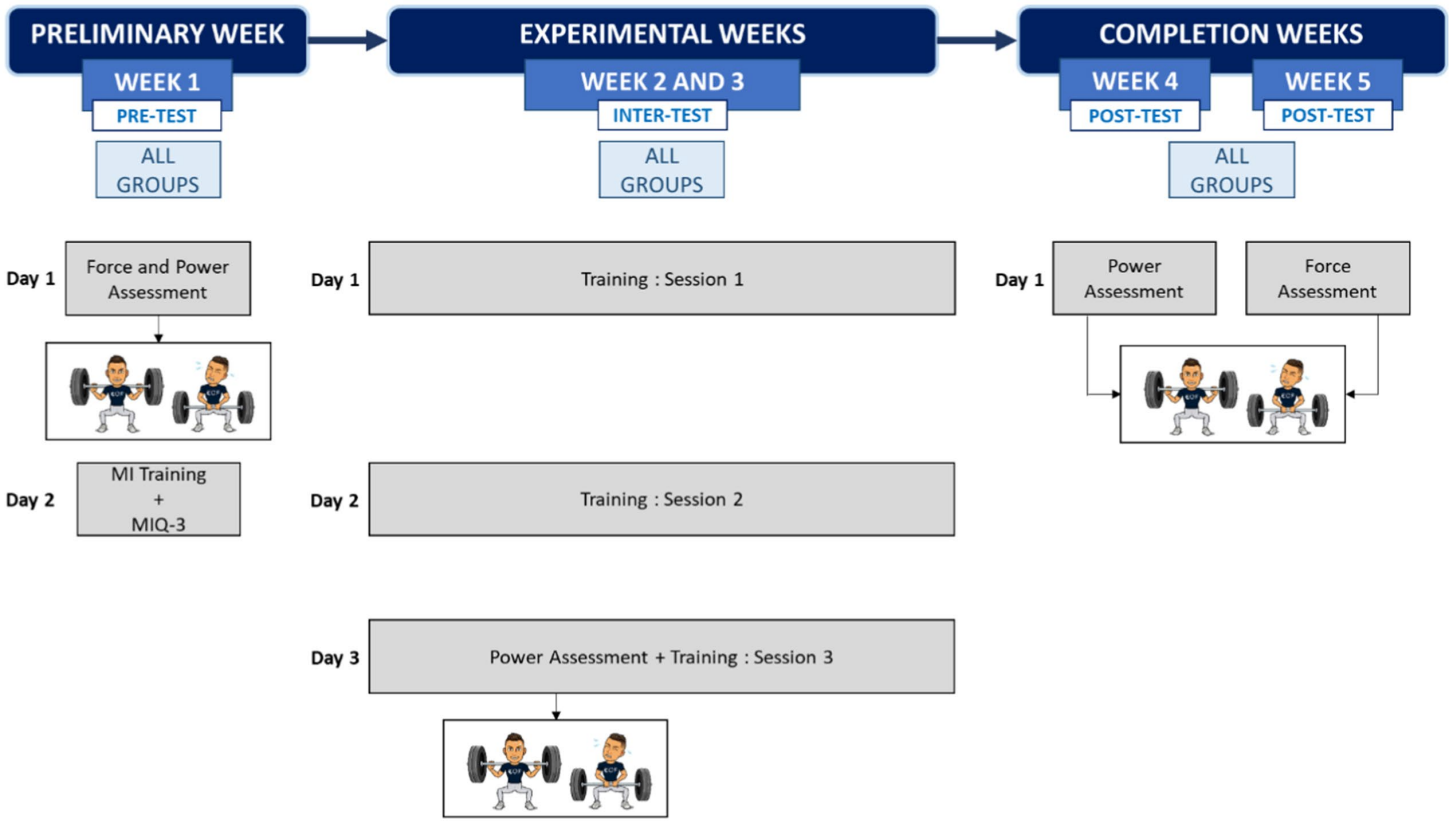
Flowchart of the experimental protocol. MIQ-3, motor imagery questionnaire.

We implemented a test-retest design, scheduled over five consecutive weeks (Figure 1). The design involved 2 weeks of training, with 3 training sessions per week. Each training session involved a combination of physical and MI training. To avoid circadian effects, the training sessions were performed at the same daytime (between 4 pm and 6 pm) and lasted 60 min. Training sessions were separated from each other by a no-training period of 48 h. Athletes trained on Monday (session 1), Wednesday (session 2) and either Friday or Saturday (session 3). The first week was dedicated to pre-test assessments of force performance. More specifically, we evaluated force and power on two exercises that involved the lower limbs and were part of official powerlifting events, i.e., the back squat and the deadlift. The second and third weeks were devoted both to the physical and MI practice interventions. We implemented intermediate evaluations of power performances (inter-tests 1 and 2). The fourth week was used as a final assessment of power (post-test power) while the fifth week was used for final assessment of strength (post-test strength). The timeline of force and power evaluations aimed at preventing carryover effects from one evaluation session to the other while gaining access to the time course of change in force performance throughout the design. Participants were randomly assigned either to one of two MI groups, or to a control (CTRL) group. During the 2 weeks of intervention, the content of the physical training (PT) was identical in the three groups. The PT program on focused back squat training. During inter-set periods, however, participants from the CTRL group were asked to perform a neutral cognitive task (looking at sports information on their smartphones) for the same amount of time as MI in the two imagery groups. One MI group mentally rehearsed the back squat, by focusing on muscle contractions of the lower limbs. The other MI group was asked to perform imaginary contractions of a distinct anti-gravitational movement that also involved the lower limbs (deadlift).

### 2.3. Strength and power training sessions

**Figure 2:**
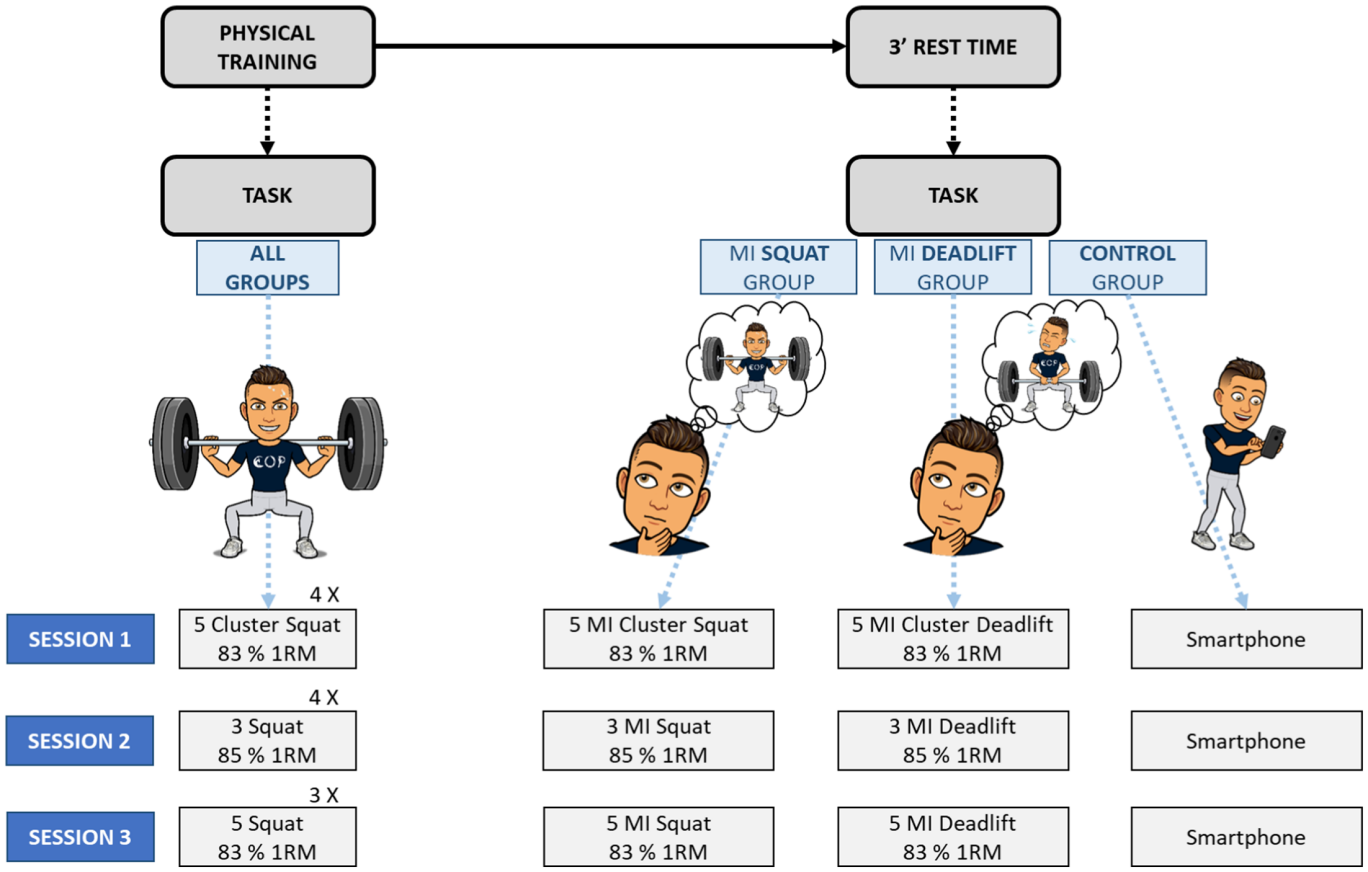
Flowchart of the training session. 1RM, one-repetition maximum; MI, motor imagery.

The workload was predetermined to avoid overtraining and limit the risk of injury. Training sessions were designed with respect to *ad-hoc* training methods targeting the development of maximum force performance. PT included exclusively back squat repetitions. During training sessions, participants performed squat repetitions at maximal speed and full range of motion. In both MI groups, mental practice was implemented during the 3 min inter-trial periods. Practically, participants were asked to combine internal visual and kinesthetic MI of the targeted movement, namely the back squat or the deadlift, depending on the experimental group. For all training sessions, the number of physical and imagined contractions was controlled, as summarized in Figure 2.

Table 1 shows the content of each training session. The volume and intensity of physical and MI training were identical each week. In order to limit the risk of injury, physical training loads were slightly reduced from 6% to 9% compared to the usual recommendations for developing maximum force (Brzycki, 1993). The three training sessions of each week were thus slightly different to avoid monotony and lassitude. Furthermore, to limit the risk of interference, physical training outside of the experimental design was carried out on upper limbs or technical skills that did not involve strength.

**Table 1:** Content of physical and motor imagery training sessions.

Session day	Volume and intensity of the training sessions	
	Physical practice	Motor imagery
1	4 sets of 5 repetitions at 83% of the pre-test	4 sets of 5 repetitions at 83% of the pre-test
2	4 sets of 3 repetitions at 83% of the pre-test	4 sets of 3 repetitions at 83% of the pre-test
3	3 sets of 5 repetitions at 83% of the pre-test	3 sets of 5 repetitions at 83% of the pre-test

### 2.4. Dependent variables

#### 2.4.1. Motor imagery ability

Before the experiment, each participant completed the revised version of the MIQ-3f in a quiet room (Table 2). The MIQ-3f is made up of 12 movements known to evaluate individual differences in visual (4 tasks using external visual imagery and 4 tasks using internal visual imagery) and kinesthetic (4 tasks) movement imagery. Data revealed high internal consistency (composite reliability scores ≥0.88 for the three subscales) and test-retest reliability (intraclass correlation coefficients of 0.87 for internal visual imagery, 0.86 for external visual imagery, and 0.88 for kinesthetic imagery) for the MIQ-3f. Completing each item required 4 steps. First, the starting position was described. Second, the movement was described for each item (either vertical knee raise, vertical jump, internal rotation of a 90° lateral arm raise, and standing toe touch stretch). Then, the participants were requested to physically perform 1 trial. Third, each individual was asked to imagine the movement from the starting position, using either visual or kinesthetic imagery as requested. Finally, each participant assigned a score on a 7-point scale regarding the ease/difficulty associated with representing each movement mentally (1 = “*Very hard to visualize/perceive*” to 7 = “*Very easy to visualize/perceive*”). The items of the questionnaire are available by request to the corresponding author.

**Table 2:** Characteristics of the athletes, imagery ability and performance scores.

GROUP	Weight (kg)	Height (cm)	MIQ-3f				SQUAT						DEADLIFT					
			IVI	EVI	KI	Total	Force (kg)		Power (Watts)				Force (kg)		Power (Watts)			
							Pre-test	Post-Test	Pre-test	Inter-Test 1	Inter-Test 2	Post-Test	Pre-test	Post-Test	Pre-test	Inter-Test 1	Inter-Test 2	Post-Test
MI SQUAT	70.48	172.04	5.96	5.95	5.49	5.8	90.68	100.88	330.08	381.39	400.08	425.19	121.52	125.24	419.38	444.28	451.27	450.88
MI DEADLIFT	70.84	173.68	6.04	6.01	5.44	5.83	90.24	97.40	331.94	382.26	396.24	403.14	121.04	128.48	438.91	474.00	496.14	503.65
CONTROL	71.16	171.2	5.88	5.95	5.42	5.75	89.36	92.80	321.54	366.62	368.25	367.78	120.04	122.08	415.43	435.23	434.38	434.20

#### 2.4.2. Repetition speed and maximal force performance

For the pre-test and the strength post-test, we collected the maximal load mobilised over five successive repetitions (5RM) for, the back squat and deadlift. Participants were allowed to complete as many trials as they wished, with a fixed delay of 5 min of passive recovery between each attempt. In order to confirm the test, the repetition speed (RS) of the fifth and final repetition was controlled and should not be above 0.30 m s^−1^. Indeed, we noted through the video that all of our athletes strongly degraded their technique below this execution speed. We therefore applied a constraint of 0.30 m s^−1^ to the last repetition to limit the risk of injury, or technique distortion. We materialized fixed horizontal and vertical points to prevent technical deviations. In particular, we used an elastic band to control the 90° knee flexion and the expected squat amplitude during each movement. During the deadlift, the elastic band was placed in the upper back to control the full extension. On the validated attempt, the load (in kg) lifted by the participants represented the maximal force.

#### 2.4.3. Maximal power

For the pre-test, inter-tests 1 and 2 and power post-test, we recorded the mean power (MP) over the 5RM, on both back squat and deadlift exercises. As muscle power is highly susceptible to fatigability, only one attempt was planned for the inter-tests 1 and 2 and the post-test. In order to ensure the reproducibility of the test, the power was always measured using the same load, namely the pre-test maximal force.

#### 2.4.4. Performance prediction

For both post-tests, we asked participants to self-determine their strength improvement on both skills (squat and deadlift) on the 5RM test. Before performing their effort, they were required to predict the load (in kg) that they assumed being able to mobilise over 5RM, meeting the technical execution criteria of the study. For the result to be conclusive, the load mobilised by the participant had to be the maximum load. If athletes were able to mobilise a higher load, they took 5 min of rest before another attempt with an additional load of 2.5 kg (which meant a better performance but a wrong performance prediction). If they were unable to complete the 5RM, the test was considered a failure. Such procedure was used to investigate whether the skill performed in MI would favour the successful prediction of subsequent maximal voluntary contraction (MVC).

#### 2.4.5. Bar velocity

During the testing periods and for both exercises, bar velocity was recorded using a linear velocity transducer (GymAware v2.10: Kinetic Performance, Canberra, Australia) for each of the 5RM. The tether device was attached to the left side of the barbell, around the widest part at the outer end. The base unit was magnetically attached to the top of a 15 kg steel plate. The device was set to be aligned directly under the bar for the duration of the two exercises. In addition, an Ipad (Apple, Inc) was placed next to the subject and recorded the performance (speed in m s^−1^ and power in Watts), using the manufacturer’s software. A camera filmed the participants in profile, during each attempt of 5RM. Video was reviewed after each attempt to confirm depth and screen technical execution of the repetition. For each repetition, data were recorded in meters per second (m s^−1^) for RS, and in Watts (W) for repetition power (RP). The 5RM of each trial were averaged for RP to assess the MP, used for analysis.

### 2.5. Statistical analyses

*A priori* sample size analysis was performed to determine how much participants were needed to achieve adequate statistical power. We used R (R Core Team, 2020) and the package pwr (Champely, 2020) to calculate the number of participants per group needed to detect a small to medium effect size, i.e., corresponding to 5%–10% of explained variation and higher, for the three-way TEST (Pretest, Posttest) × GROUP (MI_SQUAT_, MI_DEADLIFT_, CONTROL) × TASK (Squat, Deadlift) interaction tested in the random-coefficient regression model carried out on force performance measures with a statistical power of *p*_1 − *ß*_ = 0.80. Due to the repeated measures, this yielded a total of 25 participants per group, which were used as inclusion threshold.

We then used R and the package *nlme* (Pinheiro J. et al., 2020; R Core Team, 2020) to run a linear mixed effects analysis of MIQ-3f, force and power data. Accordingly, we built a series of random-coefficient regression models, with by-subject random intercepts. For MIQ-3f data, we entered the fixed effect of DIMENSION (external visual imagery, internal visual imagery, kinesthetic imagery) and GROUP (MI deadlift, MI squat, Control), with interaction term and included a by-item random intercept. To analyse force and power data, we entered the fixed effects of GROUP, TEST (Pretest, Posttest) and TASK (Deadlift, Squat), with interaction term. For power data, we entered WEEK as logarithmic numeric regressor in order to account for the expected faster improvements during early training stages (i.e., weeks 1–2) compared to late training stages (i.e., weeks 2–4). We further analysed the rate of success to achieve the predicted force level during post-test evaluations. Due to the binomial distribution of this dependent variable, we fitted a general linear mixed-effects model of logistic regression, using GROUP and TASK as fixed effects. Visual inspection of residual plots did not reveal any obvious deviations from homoscedasticity or normality. The alpha threshold for the type 1 error rate was set up at 5%. As effect sizes, we reported partial coefficients of determination (*η*^2^*_P_*) using *ad-hoc* procedure for linear mixed effects models implemented in the *effectsize* package (Ben-Shachar et al., 2020). Main and interactions effects were investigated post-hoc using general linear hypotheses testing of planned contrasts from the *multcomp* package (Hothorn et al., 2008). We applied Holms’ sequential Bonferroni corrections to control the false discovery rate (Holm, 1979).

## 3. Results

### 3.1. Motor imagery ability

MIQ-3 scores were affected by the main effect of DIMENSION [*F*(2, 819) = 24.81, *p* < 0.001, *η*^2^*_P_* = 0.06], but not by the main GROUP effect [*F*(2, 72) = 0.45, *p* = 0.63, *η*^2^*_P_* = 0.01]. Likewise, there was no GROUP × DIMENSION [*F*(4, 819) = 0.19, *p* = 0.94] interaction. Post-hoc analyses revealed that kinesthetic imagery scores [5.97, 95% CI (5.88, 6.06)] were lower compared to external visual imagery [5.96, 95% CI (5.87, 6.05)] and internal visual imagery [5.45, 95% CI (5.29, 5.61)] scores, while there was no difference between external visual imagery and internal visual imagery (*p* > 0.05).

### 3.2. Force

Force values were affected by the main effect of TEST [*F*(1, 216) = 58.28, *p* < 0.001, *η*^2^*_P_* = 0.20] and TASK [*F*(1, 216) = 1527.22, *p* < 0.001, *η*^2^*_P_* = 0.87], but not by the main GROUP effect [*F*(2, 71 = 0.15, *p* = 0.86, *η*^2^*_P_* < 0.01]. Post-hoc comparison showed that Posttest force values were greater than Pretest force values [5.66 kg, 95% CI (4.42, 6.90), *p* < 0.001]. Also, Deadlift force was superior to Squat force [29.51 kg, 95% CI (28.26, 30.74), *p* < 0.001]. The linear mixed effects analysis also revealed a two-way GROUP × TEST [*F*(2, 216) = 3.68, *p* = 0.01, *η*^2^*_P_* = 0.03] and TEST × TASK [*F*(1, 216) = 3.17, 0.03, *η*^2^*_P_* = 0.01] interaction effect. Post-hoc investigations revealed that the Pretest vs. Posttest difference in the Control group was reduced compared to that in the MI-Deadlift [4.56 kg, 95% CI (1.49, 7.63), *p* = 0.01] and MI-Squat [4.22 kg, (1.15, 7.29), *p* = 0.03] groups. Also, the Pretest vs. Posttest difference on the Squat was greater than that on the Deadlift [2.53 kg, 95% CI (0.40, 5.02), *p* = 0.04]. By contrast, the two-way GROUP × TASK interaction did not reach the statistical significance threshold [*F*(2, 216) = 1.57, *p* > 0.05, *η*^2^*_P_* = 0.02].

**Figure 3:**
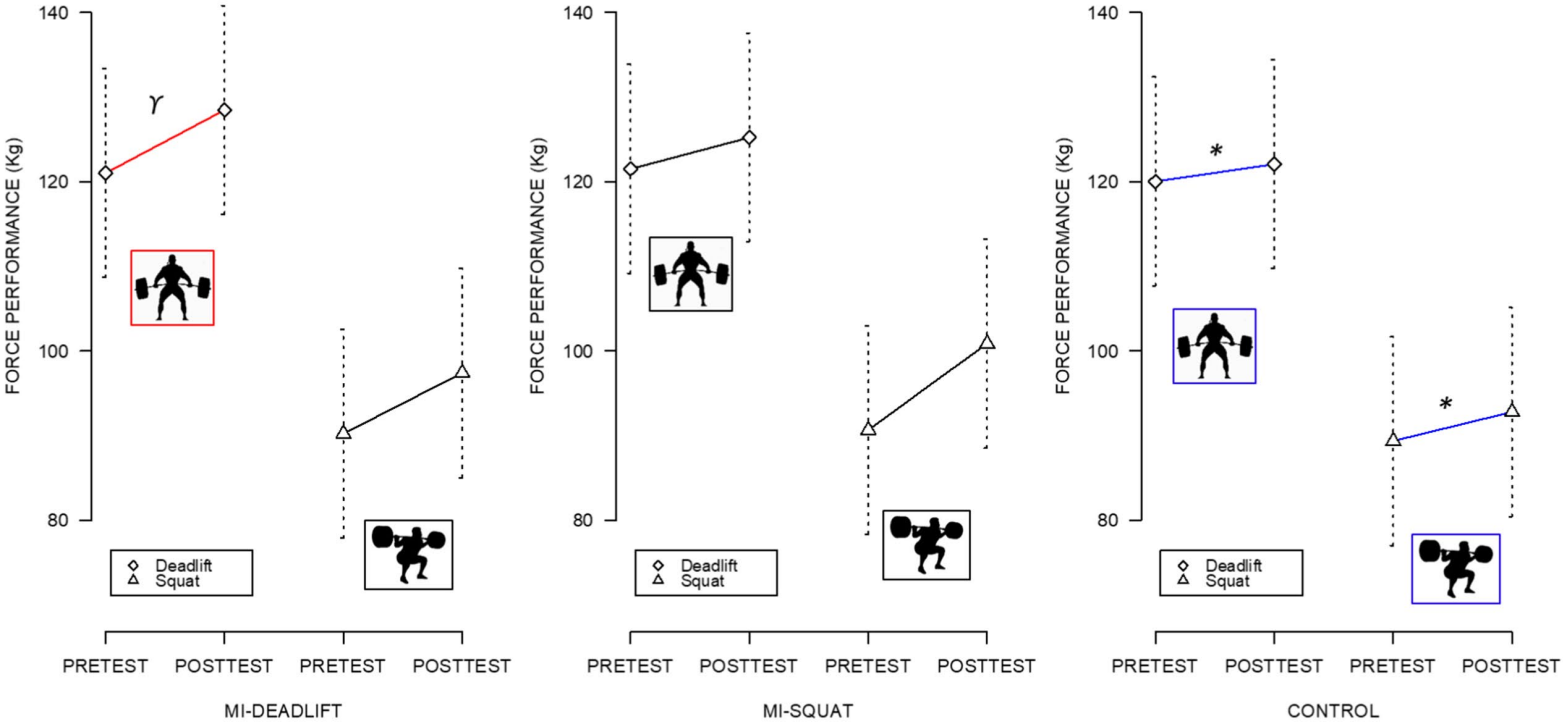
Improvement from the pretest to the posttest on squat and deadlift exercises. ϒ = greater improvement in the MI-DEADLIFT group compared to the corresponding difference in the MI-SQUAT group (*p* = 0.05). ^*^Reduced difference compared to the corresponding differences in the MI-DEADLIFT group and MI-SQUAT group (both *p* < 0.05).

A three-way GROUP × TEST × TASK interaction emerged [*F*(2, 216) = 2.25, *p* = 0.05, *η*^2^*_P_* = 0.02]. The difference in improvement from the Pretest to the Posttest between the Squat and the Deadlift in the MI-Deadlift group was higher compared to the difference in improvement from the Pretest to the Posttest between the Squat and the Deadlift in the MI-Squat group [6.76 kg, 95% CI (0.63, 12.89), p = 0.05]. This was due to reduced improvement on the Deadlift from the Pretest to the Posttest in the MI-Squat group (Figure 3).

### 3.3. Power

**Figure 4:**
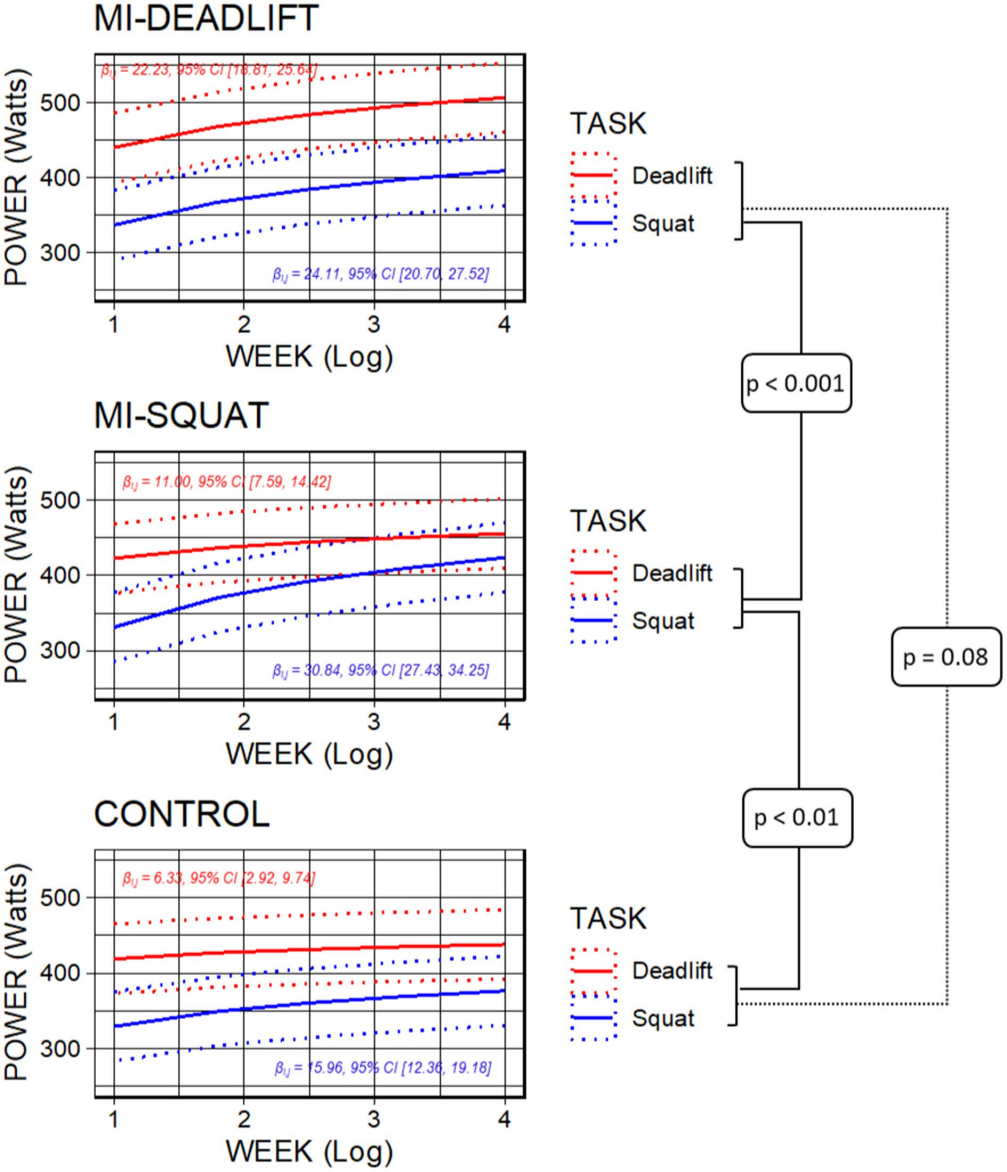
Loglinear patterns of power increase on the 5RM over the training weeks of the study. The increase pattern between squat and deadlift exercises in the MI-DEADLIFT group was identical, whereas the MI-SQUAT and CONTROL groups exhibited only greater or marginally greater improvements on the squat compared to the deadlift, respectively.

As shown by Table 3, power data were affected by the main effect of TRIAL, which corresponded to a 24.27 N Trial^−1^ (23.18, 25.38) (*p* < 0.001). The linear mixed effects analysis also revealed that power data were affected by the 3-way TEST × GROUP × TASK interaction. The power increase from weeks 1–4 between the Deadlift and the Squat in the Deadlift group was lower than that observed in the Squat group [17.95 N WEEK^−1^, 95% CI (11.12, 24.78), *p* < 0.001], while the same difference between the Deadlift group and the Control group fell short from the statistical significance threshold (7.54 N WEEK)^−1^, 95% CI (0.71, 14.38), *p* = 0.08 (Figure 4). Also, the power increase from weeks 1–4 between the Deadlift and the Squat in the Squat group was greater than that measured in the Control group [10.41 N WEEK^−1^, 95% CI (3.58, 17.24), *p* < 0.01].

**Table 3:** Summary of the ANOVA table derived from the linear mixed effects model fitted to the raw power data.

	*F*-value	*p*-value
TEST	668.55	*p* < 0.001
GROUP	0.58	*p* = 0.56
TASK	2306.29	*p* < 0.001
TRIAL	1837.19	*p* < 0.001
TEST × GROUP	27.42	*p* < 0.001
TEST × TASK	53.34	*p* < 0.001
GROUP × TASK	59.66	*p* < 0.001
TEST × GROUP × TASK	13.39	*p* < 0.001

### 3.4. Performance prediction

The ANOVA table derived from the logistic regression model carried on the success measurements during post-test evaluations was affected by the GROUP × TASK interaction [*χ*^2^(2) = 9.74, *p* < 0.01, *η*^2^*_P_* = 0.29]. There was no main effects of GROUP or TASK (*p* > 0.05). The success to achieve the Squat self-determined workload in the Squat group was higher than that the success to achieve the Deadlift self-determined workload (Table 4), whereas an opposite pattern was present in the Deadlift group (*p* < 0.01, Table 4).

**Table 4:** Success rates (conditional proportions by exercise paradigms) and number of participants to complete the exercise at the self-determined workload during the posttest.

Deadlift exercise			
Success	Deadlift	Smartphone	Squat
No	>1% (*n* = 1)	20% (*n* = 15)	12% (*n* = 9)
Yes	16% (*n* = 24)	13% (*n* = 10)	21% (*n* = 16)

Squat exercise			
Success	Deadlift	Smartphone	Squat
No	12% (*n* = 10)	18% (*n* = 14)	2% (*n* = 2)
Yes	20% (*n* = 15)	15% (*n* = 11)	30% (*n* = 23)

## 4. Discussion

The present study was designed to deepen current understandings of the effects of MI intervention on force gains. We tested whether the implementation of MI in applied settings could contribute to improve maximal voluntary strength on anti-gravitational movements. A secondary aim was to investigate, for the first time, whether MI training focusing a different movement than the one physically trained as part of the program—yet involving comparable agonists—could promote inter-task transfer of force gains. Data first revealed that participants who engaged in MI outperformed the CTRL group at post-test, hence supporting the positive effects of MI on force performance during anti-gravitational movements. Data further supported the inter-task transfer of force gains when MI targeted a movement that was not physically trained. Interestingly, participants more accurately predicted their motor performance at post-test for the exercise which was targeted by the MI training intervention only.

Participants who engaged in MI during inter-trial periods improved their force performances, which is consistent with previous studies underlying the benefits of MI training in force tasks scheduled over several weeks (for an extensive review, see Paravlic et al., 2018; Liu et al., 2023). In particular, our results corroborate observations by Lebon et al. (2010) and Reiser et al. (2011) who reported improvement of muscle strength after MI of weightlifting polyarticular and dynamic exercises. These results are somewhat challenging as greater beneficial effects of MI have been found for finger than large muscles (Liu et al., 2023). Further, there is yet no convincing evidence that the combination of MI and physical practice is more effective than conventional strength training alone (Paravlic et al., 2018). Consideration for the nature of the MI intervention enables to state hypotheses with regards to its influence on force performances over the course of several weeks of training. Reiser et al. (2011) questioned the efficacy of different ratios of physical to MI training. While the effects were more pronounced in the physical training group, all participants who trained with MI also exhibited force gains. These remained stable 1 week after the end of the experiment. The authors concluded that high-intensity strength training sessions could be partly replaced by MI without hampering their efficacy (see also Dello Iacono et al., 2021, for positive effects of MI during detraining periods). The type of imagery as well as the intensity of the imagery experience, with reference to the level of mental effort, further contributed to the beneficial effects of MI on force (Yao et al., 2013; Jiang et al., 2017). In these studies, adopting a first-person perspective along with the kinesthetic imagery modality, and training with a high mental effort combined with a low level of physical exercise, appeared more effective to improve force output during voluntary contractions. In the present study, we implemented a controlled training program for experts, to which we embedded first-person and kinesthetic imagery. Since training sessions were not decoupled from the physical training, the intervention in MI groups involved high cognitive load in addition to the physical strain of the protocol. Our present intervention can thus be considered more demanding than the frameworks adopted in previous MI studies testing its effects on force development. The combination of MI and its implementation in the context of physical training could have magnified its influence on force performances.

The main original finding of the present study is the transfer of force and power gains from one motor skill (Squat) to another one that involves the same muscles but was not trained physically (Deadlift). Combining physical practice on a given skill and MI practice of another skill involving the same agonists—yet involved in a distinct pattern of activation—facilitated performance on the secondary movement. These findings support a novel and promising effect of MI, and extend the way of how implementing MI during physical training sessions. Indeed, MI practice usually focuses the movement that is physically trained (Reiser et al., 2011; Di Rienzo et al., 2015). While the transfer of performance gains following MI has received little attention (Guillot et al., 2022), there is ample scientific evidence that strength gains observed in one task after physical practice can transfer to improvements in other tasks. First, some studies found that cross-education, which occurs when training one limb leads to strength gains in the contralateral untrained limb (e.g., Munn et al., 2004; Green and Gabriel, 2018a,b), remains effective even when the training limb is immobilized, suggesting that the transfer of strength gains is not only due to an increase in muscle mass, but also involves neural adaptations (Ruddy and Carson, 2013). Strength training has further been found to reduce the bilateral deficit with transfer of strength gains to bilateral movements (Lee et al., 2021), as well as between tasks that share similar movement patterns or muscle groups (Hendy and Lamon, 2017). The post-activation potentiation reflecting the increase in muscle force production that occurs after a maximal or near-maximal effort has also been shown to transfer to other tasks that involve similar movement patterns (McBride et al., 2005). Interestingly, present findings support that such force gains transfer can be leveraged through MI. This advocates for the potential relevance of targeting MI on another task that the one that is physically trained, provided that both motor tasks involve the activation of the same agonists. Previous experimental studies sporadically revealed that mentally training on one body side could lead to improvement in the untrained side (Yue and Cole, 1992; Bouguetoch et al., 2021), especially in case of intermanual transfer paradigms (Kohl et al., 1992; Amemiya et al., 2010; Lohse et al., 2010; Land et al., 2015; Oosawa et al., 2019). The imagery literature also supported that the benefits provided by a MI intervention might contribute to improve performance of another closed motor skill (Lejeune et al., 1994; Nicholson et al., 2018; Guillot et al., 2022). The existence of a transfer of strength gains from one task to a different one is innovative and opens the way to a new approach of science-based strength training programs. We can first postulate that mentally rehearsing the deadlift is likely to target muscles of the lower limb that are also involved, albeit for a different contraction, during the back squat. Both are anti-gravitational movements involving proximo-distal coordinations of the lower limbs, and requiring core stabilization. In line with previous work dealing with this issue, we further postulate that transferable strength gains primarily result from neural adaptations including increase in the neural drive to motor units yielding increase firing rates (Yue and Cole, 1992; Ranganathan et al., 2004; Liu et al., 2023), downregulation of presynaptic spinal inhibition (Grosprêtre et al., 2018, 2019), and/or reduction of the agonist/antagonist co-activation (Dos Anjos et al., 2022). Pascual-Leone et al. (1995) nicely demonstrated that motor imagery led to same plastic changes than physical practice of the same task. They concluded that acquisition of a motor skill through MI was associated with modulations of cortical motor outputs to the muscles involved in the task, resulting from an increase of synaptic efficacy in existing neural circuits and/or unmasking of connections due to disinhibition. Mentally rehearsing a task involving the same target muscles than another task which is physically performed might thus benefit from such plastic changes and promote inter-task transfer. As a complementary hypothesis, some authors provided evidence that in addition to neural plasticity at the cortical level, the reinforcement of synapse conductivity at the spinal level might also participate in the benefits of MI practice (Grosprêtre et al., 2016).The activation of low-threshold spinal structures might thus highlight the possible generation of subliminal cortical output during MI.

Data finally showed that the prediction of success, measured through the ability to complete the 5RM at the self-determined workload during the post-test, was higher for the exercise focused by the content of the MI intervention. Participants who imagined the back squat were able to better predict their back squat performances, whereas those who rehearsed the deadlift better predicted their performance on this specific movement—despite having trained physically on the squat. A first explanation may come from studies providing evidence that MI share the same computational processes than those involved in the modelling of voluntary movements. MI reproduces predictive operations, such as forward modelling which anticipates the sensory consequences of movement (Kilteni et al., 2018; Rieger et al., 2023). Physical and imagined sensory feedback may therefore have comparable effects on action anticipation (Pinheiro A. P. et al., 2020), hence leading to the ability to accurately predict errors and evaluate the capacity to successfully perform a task. These findings might also be explained by the motivational components of imagery (Paivio, 1985; Hall et al., 1998). Although the current MI program focused on the cognitive specific function of MI, we postulate that participants may have concomitantly strengthened both their motivation to improve performance and their self-confidence. Increasing self-confidence may then have contributed to be better prepared and conditioned to perform well, increasing the odds to reach success on self-imposed physical workload. This hypothesis is in line with the imagery literature supporting the interrelation between motivational and cognitive functions of MI (Callow et al., 2001; Short et al., 2002; Callow and Waters, 2005). Developing such ability might be of particular interest in powerlifting and weightlifting athletes, who must choose their weight strategically to achieve the better use of a limited predetermined number of attempts during competitive events. Before drawing definitive conclusions, however, future studies should replicate these findings and certainly assess the degree of self-confidence, motivation, and self-determination of athletes over the course of MI practice intervention. Practically, coaches primarily use the 1RM guideline for designing the training session and adjust loads. The ability to predict success is interesting as it allows athletes to better manage their effort within a session training and to adjust the appropriate load for the exercise. It may further contribute to readjust the load even if the athlete did not perform another 1RM, but is convinced and confident in his/her ability to well perform. Another point is that the 5RM can contribute to estimate the 1RM. In this vein, a better self-estimation of the 5RM is likely to improve the self-estimation of the 1RM as well. Altogether, this may thus help athletes to slightly and appropriately increase the load for their training without performing too many and regular 1RM tests.

The present study is not without limitations that should be considered before drawing firm conclusions. A first limit is the lack of neurophysiological measures correlative of the mental work and of the performance, which may have provided relevant information about the mechanisms involved in the inter-task transfer. Another limit is that only confirmed athletes with former experience of muscular training, and engaged in competitive activities at the national level, were included in the experimental design. Although smaller performance gains are usually observed over time in highly skilled athletes than in novices, future studies should certainly replicate and confirm the present positive effects in a sample of non-expert athletes, before generalizing the benefits of MI for inter-task transfer. Finally, and as recommended in the MI literature, we requested participants to concomitantly perform kinesthetic imagery and internal visual imagery. Recent findings however underlined the efficacy of combining the different sensory modalities (e.g., Taube et al., 2015; Eaves et al., 2016, 2023; Sun et al., 2016). Some authors also reported that offering an immersive scenario through virtual reality is likely to strengthen the benefits of kinesthetic imagery (Lakshminarayanan et al., 2023), and to create learning environments that exceed the individual’s performance level (Frank et al., 2023). Investigating different combinations of the MI experience might thus modulate the performance improvement and the inter-task transfer of force gains. Other aspects may also be viewed in perspective. The present work was designed to investigate performance on lower body strength. Both the deadlift and the backsquat are anti-gravitational movements involving proximo-distal coordinations of the lower limbs, and requiring core stabilization. This may have facilitated the inter-task transfer, and future studies should definitely test whether such effect remains effective in specific upper-limb movements involving smaller muscle groups.

## 5. Conclusion

Present results provided evidence that force gains elicited by MI might both be transferred to another different motor skill involving the same body part, and contribute to improve force performance. Spurred by these findings, new recommendations could be delivered when implementing MI into physical training sessions designed to improve force development. The use of MI during inter-trial periods could thus not only focus on rehearsing the movement that has just be performed or optimize the active/passive recovery before the next trial, but may also be designed to target performance of a secondary task to facilitate strength transfer. As further research is required to understand and elucidate the neural mechanisms underlying this phenomenon, it is likely that this approach will become an increasingly popular and represent an effective tool for athletes, coaches, and trainers seeking to maximize strength gains. These results might also allow trainers to offer less physically demanding sessions, and promote a perspective of progression on a task that could not be physical trained, due to injury or training overload risks. Taken together, and from a practical perspective, a high imagery ability seems required to successfully and appropriately perform this kind of MI. Indeed, previous research showed that, to be effective, MI must preserve the spatio-temporal features of the corresponding movement (Roure et al., 1998; Holmes and Collins, 2001; Guillot, 2020). Targeting the content of the mental representation on a secondary task that has not been performed beforehand will here require to use the sensory feedback of the physically trained movement as an emulator of the secondary task, to further be able to accurately predict its sensory feedback.

## Data availability statement

The raw data supporting the conclusions of this article will be made available by the authors, without undue reservation.

## Ethics statement

The requirement of ethical approval was waived by the local ethics committee of the university. The studies were conducted in accordance with the local legislation and institutional requirements. Written informed consent was obtained from the individual(s) for both the participation in the experiment and the publication of any potentially identifiable images or data included in this article.

## Author contributions

EP, FD, and AG: conceptualization. EP, FD, OB, and AG: methodology. EP: investigation. FDR: data analysis and writing of the result section. EP, FDR and AG: writing—original draft preparation. FD and AG: writing—review and editing and project administration. All authors contributed to the article and approved the submitted version.

## Conflict of interest

The authors declare that the research was conducted in the absence of any commercial or financial relationships that could be construed as a potential conflict of interest.

## Publisher’s note

All claims expressed in this article are solely those of the authors and do not necessarily represent those of their affiliated organizations, or those of the publisher, the editors and the reviewers. Any product that may be evaluated in this article, or claim that may be made by its manufacturer, is not guaranteed or endorsed by the publisher.
